# Placenta Growth Factor in Eyes with Neovascular Glaucoma Is Decreased after Intravitreal Ranibizumab Injection

**DOI:** 10.1371/journal.pone.0146993

**Published:** 2016-01-19

**Authors:** Minwen Zhou, Jiawei Wang, Wei Wang, Wenbin Huang, Xiaoyan Ding, Xiulan Zhang

**Affiliations:** 1 Zhongshan Ophthalmic Center, State Key Laboratory of Ophthalmology, Sun Yat-Sen University, Guangzhou, People’s Republic of China; 2 Department of Ophthalmology, Shanghai First People’s Hospital, School of Medicine, Shanghai JiaoTong University, Shanghai, China; 3 Shanghai Key Laboratory of Fundus Disease, Shanghai, China; Van Andel Research Institute, UNITED STATES

## Abstract

**Purpose:**

To evaluate changes in the concentrations of placental growth factor (PlGF) and vascular endothelial growth factor-A (VEGF-A) in aqueous humor of patients with neovascular glaucoma (NVG) before and after an intravitreal injection of ranibizumab (IVR) and to determine the underlying correlation between the levels.

**Methods:**

The prospective interventional comparative study involved 20 eyes of 20 patients with surgery-required advanced NVG and 20 control subjects from January 2013 to November 2013. The NVG eyes received the IVR treatment before glaucoma surgery. Aqueous humor was collected at the time of the IVR injection (pre- IVR) and at the time of antiglaucomatous surgery (post-IVR). Aqueous humor was also collected at the time of cataract surgery in normal control. Aqueous humor and plasma VEGF-A and PlGF levels were measured with an enzyme-linked immunosorbent assay methods, respectively.

**Results:**

The mean aqueous humor PlGF and VEGF-A concentrations in the pre-IVR eyes were significantly higher than in those of the control subjects (p<0.001), whereas the plasma levels showed no significant difference. There was a statistically significant correlation between the aqueous humor PlGF and the VEGF-A concentration (*r* = 0.612, p = 0.003). The mean aqueous humor PlGF in the post-IVR eyes dramatically decreased from 1078.36 ± 755.83 to 177.64 ± 151.73 pg/mL (p<0.001). The VEGF-A level showed a similar trend from 3697.64 ± 2104.47 pg/mL to 183.54 ± 130.35 pg/mL (p<0.001).

**Conclusions:**

Aqueous humor concentrations of VEGF-A and PlGF were significantly elevated in the eyes with NVG, and there was a positive correlation between the levels. After an IVR treatment, VEGF-A and PlGF were significantly decreased in NVG eyes.

## Introduction

Neovascular glaucoma (NVG) is an intractable, challenging disease of the eye that can result in permanent blindness. The main characteristics include iris and angle neovascularization, which almost invariably appear[1]. Angiogenesis is a key aspect of ocular neovascularization and NVG. Studies have provided substantial evidence that vascular endothelial growth factor-A (VEGF-A) is a major mediator of angiogenesis and vascular leakage in ocular neovascularization[2,3,4]. VEGF-A is a prototype member of the VEGF family, which includes placental growth factor (PlGF), VEGF-B, VEGF-C, VEGF-D, and VEGF-E[5]. The importance of VEGF-A in hypoxia-induced neovascularization is well known, but the role of the other recognized members of the VEGF family of peptides in ocular neovascularization and their interaction with VEGF-A is less well-defined.

PlGF is a member of the VEGF family of angiogenic molecules[6]. Many cell types produce PlGF, especially when activated or under stress[7]. Studies have shown that it is involved in endothelial stimulation, pathological angiogenesis, and wound healing[7,8] and that it directly stimulates neovascularization by inducing the proliferation, migration, and survival of endothelial cells [8]. Study demonstrated that PlGF alone enhances angiogenesis through the activation of VEGF receptor type 1 (VEGFR1)[8].

In recent years, a number of studies have focused on the role of PlGF in the pathogenesis of ocular neovascularization[9,10]. Mitamura et al. found that PlGF levels were significantly higher in active proliferative diabetic retinopathy[11]. Another study found that PlGF mRNA expression was significantly up-regulated during the course of experimental choroidal neovascularization (CNV) and demonstrated the participation of PlGF in experimental CNV[10]. However, little has thus far been uncovered regarding the involvement of PlGF in NVG patients.

As VEGF-A blockade has been demonstrated to efficiently reduce neovascularization progression and leakage in ocular neovascular disease, various VEGF-A inhibitors have been clinically developed[12,13,14]. Among these, ranibizumab is a high-affinity recombinant Fab, which neutralizes all isoforms of VEGF-A[15]. Wang et al. found that an intravitreal injection of ranibizumab (IVR) can significantly decrease aqueous VEGF-A concentrations in eyes with age-related macular degeneration [16]. Another study showed that PlGF and VEGF-A belong to the VEGF family of angiogenic molecules and that PlGF has 42% amino acid sequence identity with VEGF-A[17]. However, to date, little is known about the potential involvement of PlGF in NVG and the change in PlGF concentrations in eyes with NVG following treatment with IVR.

In this study, we examined VEGF-A and PlGF levels in the aqueous humor and the plasma of patients with NVG and compared these before and after an IVR (pre-IVR and post-IVR, respectively).

## Methods

### Subjects and enrolment criteria

This prospective and comparative study included patients with NVG who attended the hospital in the study period from January 2013 to November 2013. The study protocol complied with the provisions of the Declaration of Helsinki and was approved by the Ethical Review Committee of Zhongshan Ophthalmic Center. Written informed consent was obtained from all the participants involved in the study. All the patients underwent a complete ophthalmic examination, including best-corrected visual acuity, indirect stereoscopic ophthalmoscopy, measurements of intraocular pressure (IOP) by Goldmann tonometer, and slit-lamp biomicroscopy.

All patients who presented with neovascularization on the iris and the anterior chamber angle and had an established diagnosis of NVG first received the IVR treatment. NV eyes were enrolled and only those who had persistently elevated IOP a week later and who required surgery were included in the current sample and scheduled for antiglaucomatous FP-7 Ahmed glaucoma valve implantation (New World Medical Inc., Rancho Cucamonga, CA, USA). The medications for decreasing the IOP were applied as follows: topical β-blockers, topical carbonic anhydrase inhibitors, and topical α2-adrenergic agonists. Systemic medications to decrease the IOP were applied if necessary. The exclusion criteria for NVG were: (1) systemic conditions potentially affecting VEGF-A and PlGF levels, except hypertension and diabetes mellitus, (2) unable to provide informed consent, (3) pregnant woman, and (4) unable to tolerate the operation. The control group included the same exclusion criteria as the NVG group, in addition to prior intraocular surgery, the presence of any other ocular diseases, hypertension, and diabetes mellitus. The only systemic medications allowed were those used for arterial hypertension and for diabetes mellitus in the NVG group.

### Anti-VEGF injection

As our previous study described[2], all the NVG patients received 0.5 mg of an IVR using a 30-gauge needle in the inferior- temporal quadrant at 3.5 mm to 4 mm posterior to the limbus. The antiglaucomatous medications (introduced above) and an ophthalmic suspension of anti-inflammatory prednisolone acetate 1% eye drops (Allergan, Inc. Irvine, USA) were applied for controlling the IOP and the inflammatory response.

### Aqueous humor sampling

The aqueous humor samples were obtained at two time points (as our previous study described[2]): 1) at the time of the IVR administration (pre-IVR) and 2) at the time when the sequential Ahmed glaucoma valve implantation was performed (post-IVR). Aqueous humor was collected by means of anterior chamber paracentesis performed with a 30-gauge needle and the volume of the aspirated aqueous humor samples was about 100μL. Each aqueous humor sample was then placed into an Eppendorf tube, rapidly frozen at -80°C, and protected from light until the levels of VEGF-A and PlGF were measured.

Aqueous humor samples were also collected from 20 eyes (20 patients) of the healthy controls at the time of cataract surgery (phacoemulsification plus intraocular lens implantation) at the Glaucoma Department of Zhongshan Ophthalmic Center from January 2013 to November 2013. All the samples were obtained at the beginning of the surgery prior to any conjunctival or intraocular manipulation to avoid breakdown of the blood—aqueous barrier associated with surgical trauma.

### Blood sampling

As our previous study described[2], blood samples (5 mL) were taken just prior to the cataract surgery or the treatment with the IVR, placed on ice, and immediately brought to the laboratory where they were centrifuged for 15 min at 1000 g and 4°C. The supernatant plasma was then formed into aliquots and stored at -80°C until they were assayed.

### VEGF-A and PlGF analyses

The VEGF-A and PlGF were measured in the aqueous samples from the same eye, as well as in the plasma. The concentrations of VEGF-A and PlGF were measured by a VEGF-A ELISA kit (Biovendor, Cat. No.: RBMS277/2R, Modrice, Czech Republic) and a PlGF ELISA kit (DRG, Marburg, Germany) according to the manufacturer’s protocol and as described elsewhere[2,18,19]. In brief, 20 μl of each sample was pipetted into 96 well plates pre-coated with polyclonal anti-human VEGF-A/PlGF antibody. After incubation at room temperature, the wells were emptied and washed several times with the washing buffer provided. After incubation with a second antibody, any excess antibody was washed off and samples were incubated with substrate in the dark. The reaction was terminated by adding a stop solution to each well. The absorbance of the resulting yellow product was measured at 450 nm using a multiwell plate reader (Multiskan Ascent; Thermo Fisher Scientific GmbH, Schwerte, Germany). A standard curve was prepared by plotting the absorbance values against the concentrations of standards, and the concentrations of any unknown samples were confirmed based on this standard curve. All the samples were prepared and detected on the same day with the same methods by the same experimental technician.

### Data analysis

Statistical comparisons of the two groups were done using an independent sample *t* test for normally distributed continuous variables. When the latter criteria did not apply, a Mann—Whitney *U* test was performed. Categorical covariates were assessed individually with Fisher’s exact test. The Wilcoxon-signed rank test was used to detect changes pre-IVR and post-IVR within the groups. Spearman’s rank-order correlation analysis was used to analyze the correlation between the aqueous humor PlGF and the VEGF-A in the NVG patients, and the correlation between the aqueous humor and the plasma PlGF and VEGF-A concentrations in both groups. Multivariate logistic regression analysis was used to evaluate the potential factors associated with the presence of NVG. All statistical analyses were performed using SPSS 13.0 for Windows (SPSS Inc., Chicago, IL). For all tests, p<0.05 was considered significant. The data are presented as mean ± standard deviation (SD).

## Results

### Patients’ demographic data

Twenty-nine NVG patients were screened in this study. Of these patients, four had to be excluded because too little aqueous humor could be obtained at the time of the IVR administration. Five patients were excluded because they did not need antiglaucomatous surgery after receiving the IVR treatment. Finally, a total of 20 patients (20 eyes) with NVG who fulfilled the inclusion criteria were recruited in this study, which included two case of secondary Eales’ disease, two cases of secondary proliferative retinal detachment, two cases of unestablished reasons, five cases of secondary diabetic retinopathy, and nine cases of secondary ischemic central retinal vein occlusion. A normal control group of 20 subjects (20 eyes) who fulfilled the inclusion criteria were also included in the study. All measurements were performed successfully, and there were no failures in determining the VEGF-A or PlGF due to insufficient sampling. The mean age of the NVG patients and the normal control individuals was 52.60 ± 21.65 (mean ± standard deviation) and 60.90 ± 5.85 years, respectively. All the data are summarized in Table 1. There were no significant differences in sex or the mean age between the two groups. The time between the IVR and the antiglaucomatous surgery was 6.90 ± 1.40days.

**Table 1:** Clinical characteristics in study subjects.

Variables	NVG	Control	*P*-Values
Numbers	20	20	-
Age (SD), yrs	52.60 ± 21.65	60.90 ± 5.85	0.112 ^a^
Gender (male/female)	15/5	9/11	0.105 ^b^
IOP	45.70 ± 6.91	13.60 ± 2.52	*P*<0.001^a^
Hypertension (%)	8(40%)	0	-
Diabetes Mellitus(%)	5(25%)	0	-

NVG: neovascular glaucoma; SD: standard deviation; IOP: intraocular pressure.

^a^Independent sample *t* test.

^b^Fisher’s exact test.

### VEGF-A levels at Pre-IVR in aqueous and plasma samples

**Fig 1:**
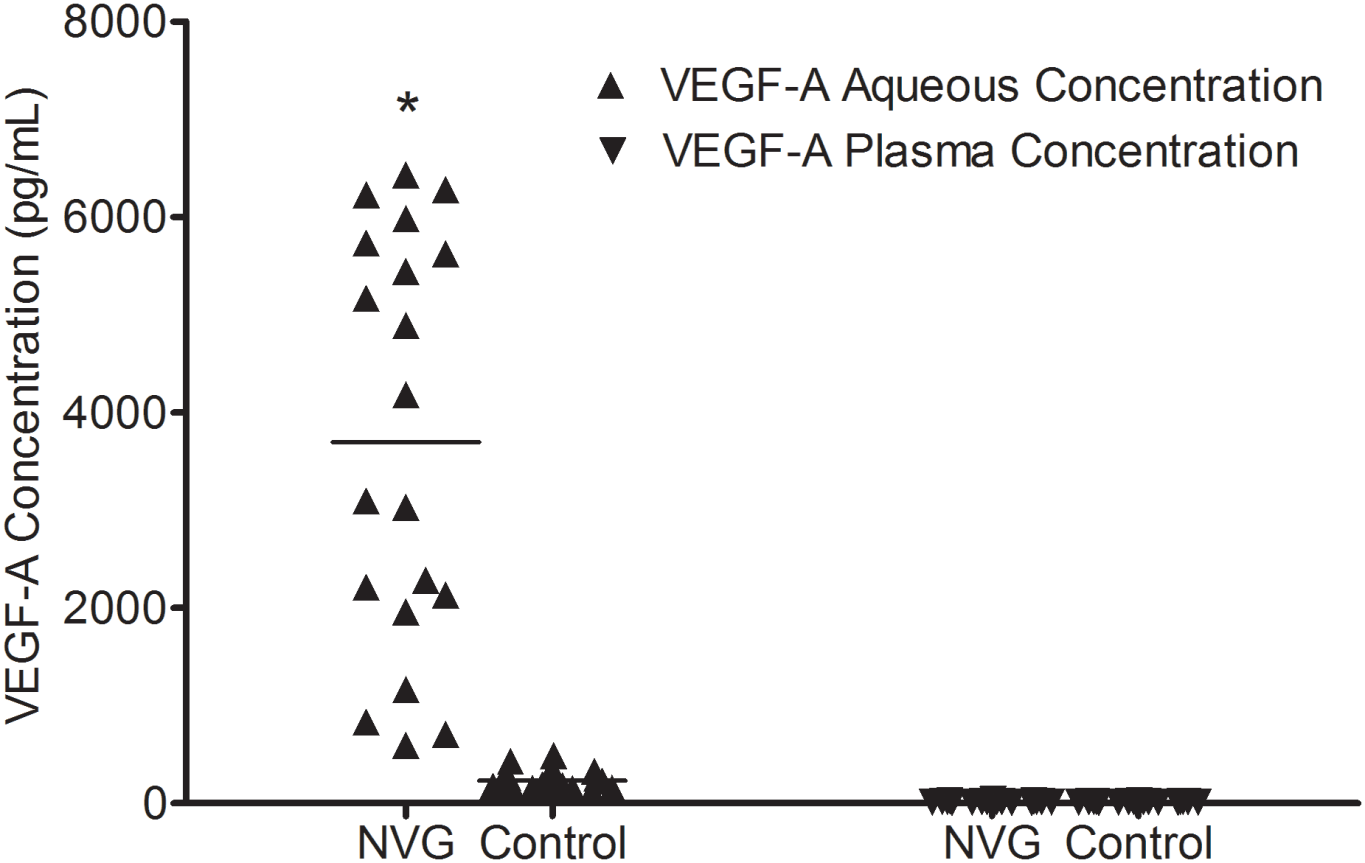
VEGF-A levels of the aqueous humor of the groups. The VEGF-A levels of the aqueous humor were significantly different between the groups (*p<0.001), whereas there was no significant difference in serum VEGF-A concentrations between the groups (p = 0.636).

The aqueous levels of pre-IVR VEGF-A were significantly elevated in the patients with NVG (3697.64 ± 2104.47 pg/mL) (median, 3634.64 pg/mL; range, 594.52–6430.17 pg/mL) compared with the control subjects (233.31 ± 97.67 pg/mL) (median, 231.94 pg/mL; range, 105.92–488.73 pg/mL) (p<0.001). However, there was no significant difference in the plasma VEGF-A concentrations between the NVG (17.76 ± 7.25 pg/mL) (median, 16.85 pg/mL; range, 8.08–39.65 pg/mL) and the control group (16.10 ± 4.27 pg/mL) (median, 15.86 pg/mL; range, 8.13–22.96 pg/mL) (p = 0.636) (Fig 1).

### PlGF Levels at Pre-IVR in aqueous and plasma samples

**Fig 2:**
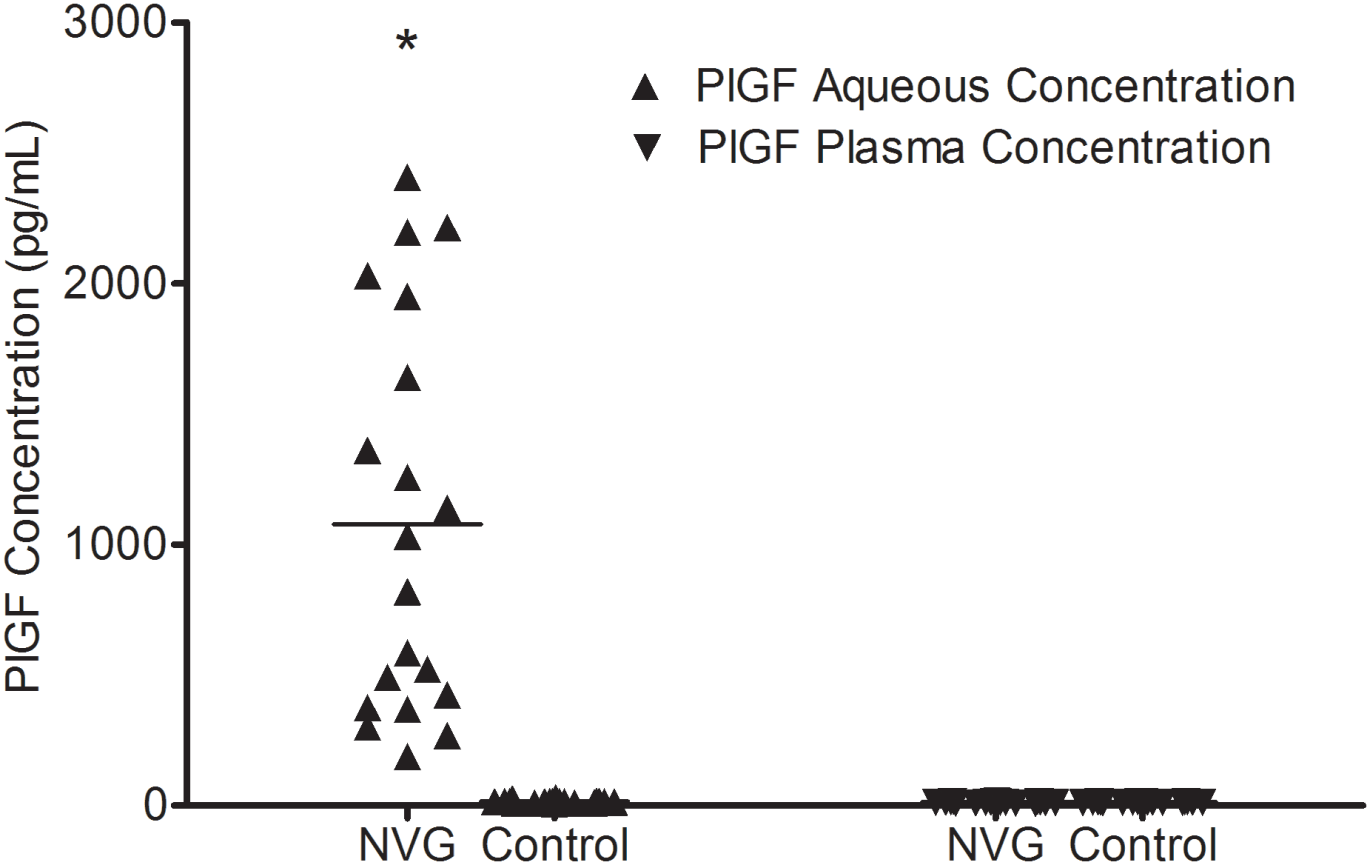
PlGF levels of the aqueous humor. The PlGF levels of the aqueous humor were significantly different between the groups (*p<0.001), whereas there was no significant difference in serum PlGF concentrations between the groups (p = 0.115).

In the patients of the NVG group, the aqueous humor concentration of PlGF at baseline was 1078.36 ± 755.83 pg/mL (median, 924.99 pg/mL; range, 186.81–2407.62 pg/mL), which was significantly (p<0.001) higher than that in the control group (12.82 ± 2.61 pg/mL) (median, 12.73 pg/mL; range, 8.77–17.35 pg/mL). The differences in the plasma PlGF concentrations between the NVG (15.70 ± 5.23 pg/mL) (median, 15.51 pg/mL; range, 8.04–30.80 pg/mL) and the control group (13.72 ± 1.32 pg/mL) (median, 13.94 pg/mL; range, 11.61–15.51 pg/mL) (p = 0.115) were not statistically significant (Fig 2).

### Correlation between the aqueous humor PlGF and VEGF-A levels in the NVG patients

**Fig 3:**
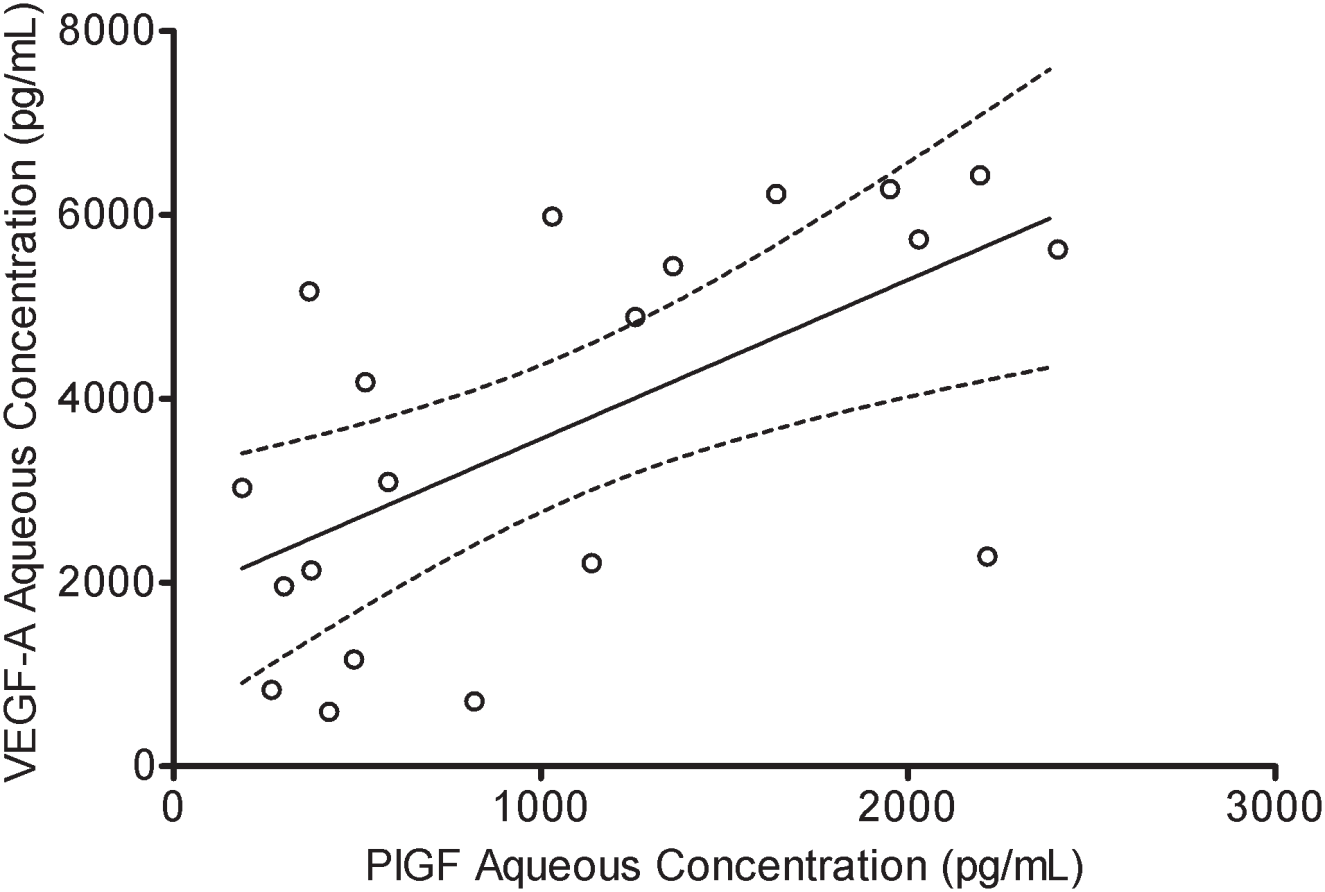
Correlation between the level of VEGF-A and PlGF in the aqueous humor. The level of VEGF-A in the aqueous humor was significantly correlated with the level of PlGF (r = 0.632, p = 0.003).

In the NVG group, the pre-IVR aqueous humor concentrations of PlGF and VEGF-A were significantly (*r* = 0.632, p = 0.003) correlated with each other (Fig 3). The result showed that there was no significant correlation between post-IVR aqueous humor concentrations of PlGF and VEGF-A(*r* = 0.236, p = 0.398).

### Multiple linear regression analysis

In the multivariate logistic regression analysis, the adjusted ORs for the NVG eyes compared with normal eyes were summarized in Table 2. Multivariate logistic regression analysis showed that neither VEGF-A nor PlGF is significantly association with NVG. In this model, the Akaike Information Criterion (AIC) value was 8.02. Since the PLGF and VEGFA were positively correlated, we removed the VEGFA from the model. After removing the VEGF-A, the AIC value changed to 6.02. As a smaller AIC value led to a better model, a new model was built again in our study. The final model includes the IOP and PLGF factors, and the results are shown that PlGF is not significantly association with NVG (Table 3).

**Table 2:** Multivariate analysis of risk factors associated with NVG.

Factors	Multivariate adjusted OR (95%CI)	*P*
IOP	0.543 (0.062, 4.791)	0.583
PlGF	0.995 (0.895, 1.105)	0.919
VFGF-A	1.001 (0.969, 1.034)	0.967

NVG: neovascular glaucoma; OR: odds ratio; CI: confidenceInterval; IOP: intraocular pressure; PlGF: placenta growth factor; VEGF-A: vascular endothelial growth factor-A.

**Table 3:** Multivariate analysis of risk factors associated with NVG.

Factors	Multivariate adjusted OR (95%CI)	*P*
IOP	0.541 (0.058, 5.028)	0.588
PlGF	0.997 (0.960, 1.034)	0.857

NVG: neovascular glaucoma; OR: odds ratio; CI: confidence Interval; IOP: intraocular pressure; PlGF: placenta growth factor.

### VEGF-A and PlGF levels at pre- and post-IVR in the NVG patients

**Fig 4:**
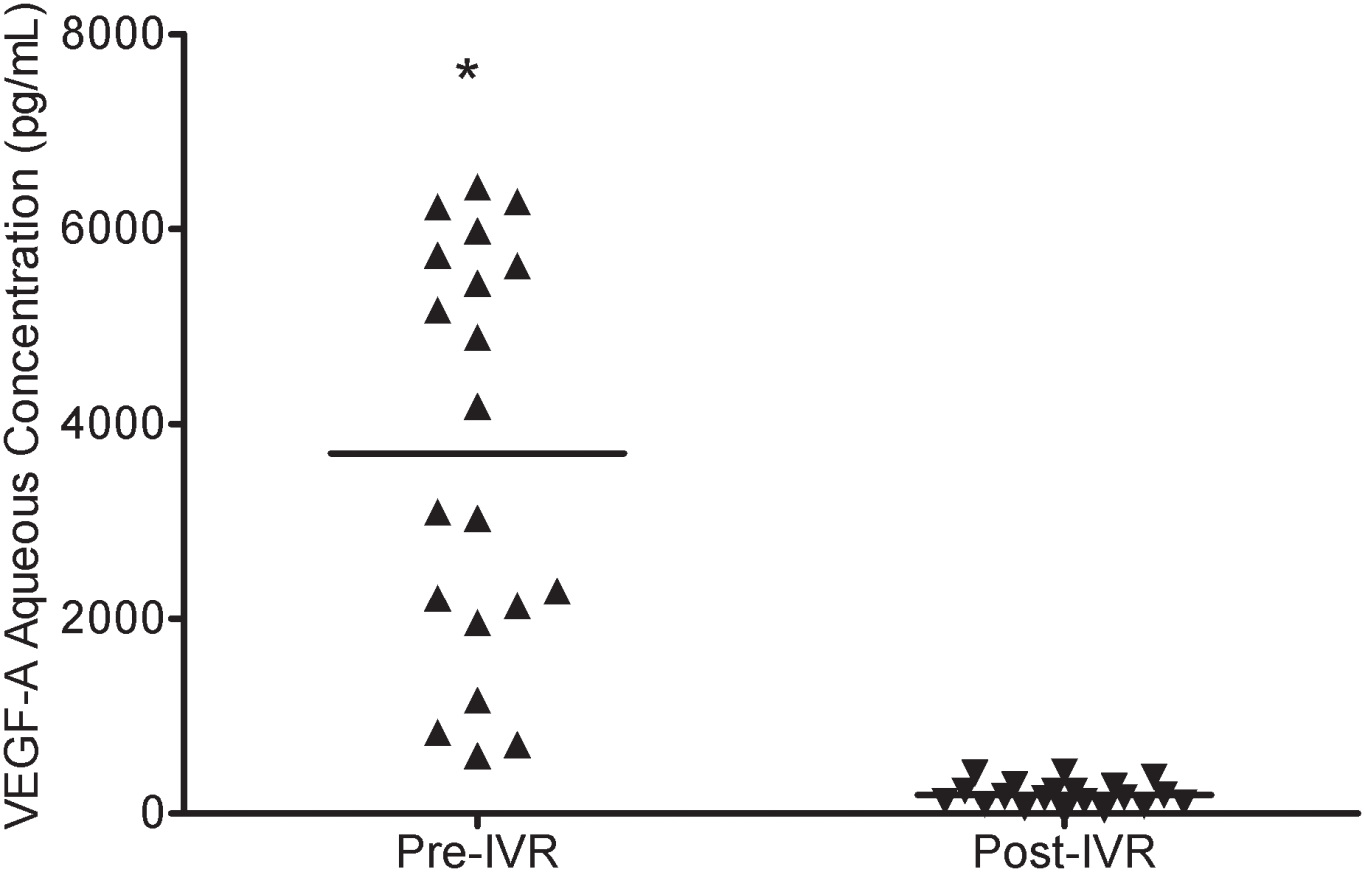
Aqueous humor VEGF-A concentration in the pre-IVR and post-IVR NVG eyes. Compared to pre-IVR, the mean aqueous humor VEGF-A concentration significantly decreased post-IVR in the NVG eyes (*p<0.001). Each point represents a measurement from a single patient.

**Fig 5:**
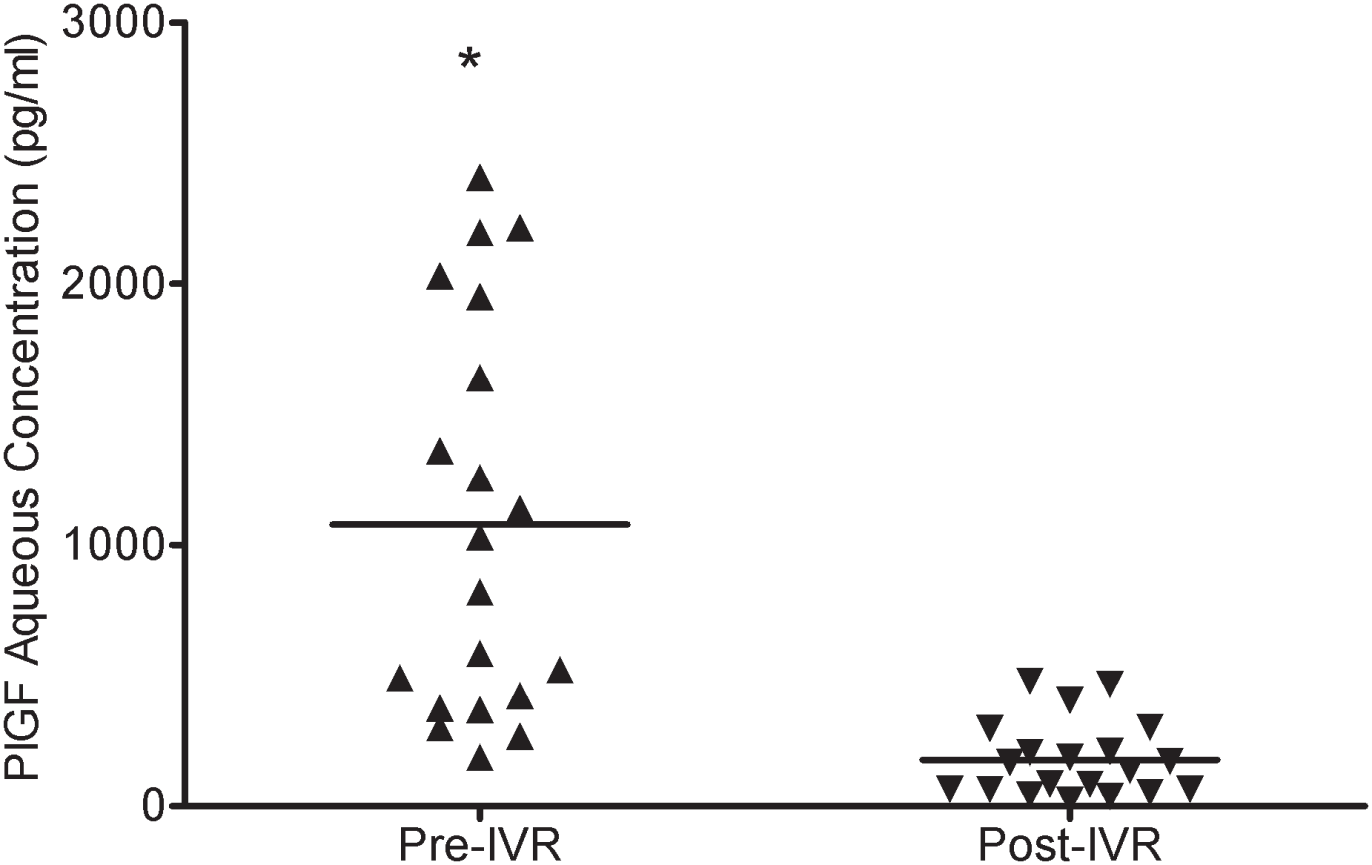
PlGF concentration pre and post-IVR. There were statistically significant differences pre- and post-IVR in the PlGF concentration in the aqueous humor (p<0.001). Each point represents a measurement from a single patient.

Compared with pre-IVR, the mean aqueous humor VEGF-A concentration (3697.64 ± 2104.47 pg/mL) (median, 3634.64 pg/mL; range, 594.52–6430.17 pg/mL) was significantly decreased post-IVR in the NVG eyes (183.54 ± 130.35 pg/mL) (median, 155.34 pg/mL; range, 12.68–429.33 pg/mL) (p<0.001) (Fig 4). The mean aqueous humor PlGF concentration pre-IVR in the NVG group was 1078.36 ± 755.83 pg/mL(median, 924.99 pg/mL; range, 186.81–2407.62 pg/mL). Post-IVR, the level decreased to 177.64 ± 151.73 pg/mL (median, 111.74 pg/mL; range, 22.70–478.79 pg/mL). The difference in the aqueous humor PlGF concentration pre- and post-IVR was statistically significant (p<0.001) (Fig 5).

### Correlations of aqueous humor and plasma VEGF-A and PlGF levels in the study groups

There were no significant correlations between the aqueous humor and the plasma VEGF-A levels in the two groups (all p>0.05). The correlations between the aqueous humor and the plasma PlGF also did not reach statistical significance in the groups (all p>0.05) (Table 4).

**Table 4:** Correlations of aqueous humor and plasma VEGF-A and PlGF levels in study groups.

Correlation	*P*-value	*r*
NVG		
Aqueous humor VEGF-A—plasma VEGF-A	0.556	-0.140
Aqueous humor PlGF—plasmaPlGF	0.535	-0.147
Control		
Aqueous humor VEGF-A—plasma VEGF-A	0.349	-0.221
Aqueous humor PlGF—plasmaPlGF	0.806	-0.059

VEGF-A: vascular endothelial growth factor-A; PlGF: placenta growth factor;NVG: neovascular glaucoma.

### Subgroup analysis of NVG group

To test whether hypertension in the NVG group affected the PlGF and VEGF-A concentrations in the aqueous humor, the PlGF and VEGF-A levels were compared between the patients with hypertension (*n* = 8) and the patients without hypertension (*n* = 12).There was no statistical difference in the PlGF and VEGF-A levels between these two groups (p = 0.643 and p = 0.537, respectively) in the Mann—Whitney *U* test. To test whether diabetes mellitus in the NVG group affected the PlGF and VEGF-A concentrations in the aqueous humor, the PlGF and VEGF-A levels were also compared between the patients with diabetes mellitus (*n* = 8) and the patients without diabetes mellitus (*n* = 12). The results were similar, with no difference in the mean PlGF and VEGF-A concentrations detected between the groups (all p>0.05) (Table 5).

**Table 5:** Subgroup analysis of NVG group.

Variables	With Hypertension (n = 8)	Without Hypertension (n = 12)	*P*-value	With DM (n = 5)	Without DM (n = 15)	*P*-value
PlGF	1185.49 ± 823.10	1006.94 ± 736.00	0.643	1284.57 ± 1015.38	1009.63 ± 678.60	0.694
VEGF-A	3131.84 ± 2255.71	4074.85 ± 2005.92	0.537	4420.82 ± 1793.93	3456.59 ± 2200.45	0.407

NVG: neovascular glaucoma; DM: diabetes mellitus; PlGF: placenta growth factor; VEGF-A: vascular endothelial growth factor-A.

## Discussion

The significance of our observations is that PlGF was significantly elevated in the NVG patients, suggesting that PlGF might have a potent angiogenic function in the induction of iris neovascularization, and that the increase in PlGF levels paralleled the increase in VEGF-A levels. VEGF-A was found to be a primary angiogenic factor, with its expression induced by hypoxia-inducible factor-1 (Hif1α) in a hypoxia-dependent fashion[20]. The marked increase in the VEGF-A concentration in the aqueous humor of the eyes with NVG in the present study is unsurprising. It has been shown in a large number of studies, including our previous research[2,4,19]. However, our study found that the PlGF concentration in the aqueous humor is also higher in patients with NVG than in healthy individuals. This finding points to significant up-regulation of constitutive ocular PlGF production in NVG. As is well known, NVG is caused by several ocular ischemic diseases, such as diabetic retinopathy, ischemic central retinal vein occlusion, and central retinal artery occlusion. A previous study also found that the expression of PlGF is up-regulated in choroidal neovascularization disease[10]. Taken together, the results of this study suggest that PlGF might have a cooperative role with VEGF-A in the progression of this hypoxia-induced disease.

Our second key finding is that the increased PlGF levels in the pre-IVR NVG aqueous humor were correlated with higher levels of the angiogenic growth factor VEGF-A. Based on our results, it is not possible to determine whether there is a causal relation between the higher concentrations of PlGF in the aqueous humor and the elevated level of VEGF-A in the aqueous humor. It is reasonable to propose that as members of VEGF family, this hypoxia-induced disease simultaneously stimulated both the up-regulation of PlGF and VEGF-A in the NVG eyes. This notion is based on the observation that PlGF and VEGF-A share a common pathway, with their expression induced by Hif1α in a hypoxia-dependent fashion[21].Studies have shown that when the retina suffered oxygen-induced ischemic retinopathy, which results in elevated HIF-1 levels,HIF-1 up-regulates several vasoactive gene products including VEGF, PlGF, and EPO[22,23,24]. When the eyes with ischemic retinopathy received the anti-VEGF treatment, it might have alleviated the condition of ischemic retinopathy and the HIF-1 levels may have decrease. Accordingly, the vasoactive gene such as VEGF, PlGF, and EPO might have decreased. In this study, the decreased level of PlGF after receiving the anti-VEGF treatment proved this theory again. However, our previous study showed that levels of EPO did not change after the anti-VEGF treatment. We assumed that it might have different signal transduction pathways and different mechanisms. Another possible explanation is that PlGF is VEGF-A dependent and that it is up-regulated by VEGF-A. However, when receiving the IVR treatment, the PlGF and the VEGF-A aqueous humor concentration lost the correlation. Further study is required to determine the exact cause of this. In addition, in the present study, we found that the aqueous levels of pre-IVR, VEGF-A, and PlGF were significantly elevated in patients with NVG, compared to the control subjects. However, multiple linear regression analysis showed that VEGF-A and PlGF are not independent risk factors for NVG. The most probable cause for this difference could be the small sample size, which included only 20 samples. This could have affected whether or not the two factors are predictors of risk for incidence of NVG. However, the multiple linear regression analysis told us that if PlGF elevated 1 pg/mL, the risk of an incidence of NVG increases by 3/1000, and if the PlGF elevated 100 pg/mL, the risk of an incidence of NVG would increases by 40%. For an exact conclusion, the sample size of the study must be increased.

VEGF-A is the first and most studied member of the VEGF family, and it is currently a key target for antiangiogenic therapy. Ranibizumab, a VEGF-A antagonist, binds with high affinity to VEGF-A isoforms. The binding of ranibizumab to VEGF-A prevents the interaction of VEGF-A with its receptors (VEGFR1 and VEGFR2) on the surface of endothelial cells, reducing endothelial cell proliferation, vascular leakage, and new blood vessel formation[25]. Currently, treatment with an IVR is effective and widely used to treat ocular neovascular disease[26,27]. In this study, using the IVR as the first step to treat NVG showed a surprising result. As we expected, we found a significant response to the IVR treatment, with the VEGF-A concentration in the aqueous humor decreasing to a dramatically low level. This was similar to the result of our previous study[2]. However, it was surprising that the PlGF concentration also decreased to a dramatically low level after the IVR treatment. It is important to note that current anti-VEGF strategies do not directly affect PlGF signaling, as Papadopoulos et al. reported[28]. In theory, VEGF-A inhibition could lead to an increase in PlGF via enhanced Hif1α feedback or via a reduction in PlGF as a result of a VEGF-dependent factor. Based on the reduced level of PlGF after the IVR treatment, we hypothesize that VEGF-A up-regulation is an important regulator of PlGF. In fact, this notion was supported by Yang’s group who reported PlGF-mediated modulation of tumor angiogenesis by a VEGF-A-dependent mechanism[29].

To elucidate whether PlGF and VEGF-A in the aqueous humor originate from the blood or are locally produced, their concentrations in aqueous humor and plasma were compared. No significant differences were found in the plasma levels of PlGF and VEGF-A between the NVG patients and the control subjects. In addition, a correlation analysis revealed no correlation between the aqueous levels and the plasma levels of the VEGF-A and PlGF in either group. These results point to local PlGF and VEGF-A production in the eye as opposed to transudation of PlGF and VEGF-A from the serum into the vitreous. Instead, PlGF and VEGF-A are likely the result of increased local production in the retina, followed by leakage into the anterior chamber.

This study took specific steps to eliminate potential inaccuracy. However, the NVG group included diabetes mellitus and hypertension patients, whereas the control group did not. The not fully matched diabetes mellitus, and hypertension might have affected the concentrations of VEGF-A and PlGF in the aqueous humor. To test whether these factors affected the level of these two factors in the aqueous humor, we performed a subgroup analysis. The validity of our findings was strengthened by the results of a relative analysis.

Nevertheless, the interpretation of our results is limited by a number of factors. First, the antiglaucomatous drugs used in the treatment of NVG might have affected the levels of PlGF and VEGF-A concentrations in the aqueous humor. Second, in the present result, we found that VEGF and PlGF levels in NVG patients are correlated. However, it is unclear whether this relationship is co-dependent. Do PlGF levels change because VEGF levels do? Or are PlGF levels independent of VEGF levels? According to the present data, we could not explain the exact mechanism of the observed phenomenon. To determine the pathogenesis of PlGF in NVG, further studies are warranted concerning the local presence and intraretinal expression of PlGF in eyes. Third, although we found that there was significant difference between the two groups, the variation between individuals was large. NVG is a complex disease; there might be various mechanisms involved in it. Therefore, this conclusion should be interpreted with caution. Finally, the sample size of this study was relative small; however, the difference in the levels of PlGF and VEGF-A between the NVG group’s and the control group’s eyes was very large, which supports the robustness of the results.

In conclusion, our study is the first to report that PlGF and VEGF-A levels are elevated in eyes with NVG. The increased PlGF levels were positively correlated with higher levels of VEGF-A in the aqueous humor of the NVG eyes. After an IVR treatment, VEGF-A and PlGF levels were significantly decreased in NVG eyes. However, the results should be interpreted with caution. In future, further studies are needed to elucidate the role of PlGF in the pathogenesis of NVG.

## Supporting Information

S1 Dataset Data of the Experiment. (XLSX)

## References

[1] Weiss DI, Shaffer RN, Nehrenberg TR (1963) Neovascular gluacoma complicating carotid-cavernous fistula. Arch Ophthalmol 69: 304–307. 13999715 10.1001/archopht.1963.00960040310007

[2] Zhou M, Chen S, Wang W, Huang W, Cheng B, Ding X, et al (2013) Levels of erythropoietin and vascular endothelial growth factor in surgery-required advanced neovascular glaucoma eyes before and after intravitreal injection of bevacizumab. Invest Ophthalmol Vis Sci 54: 3874–3879. 10.1167/iovs.12-11507 23674760

[3] Abcouwer SF (2013) Angiogenic Factors and Cytokines in Diabetic Retinopathy. J Clin Cell Immunol Suppl 1. 10.4172/2155-9899 PMC3852182 24319628

[4] Tripathi RC, Li J, Tripathi BJ, Chalam KV, Adamis AP (1998) Increased level of vascular endothelial growth factor in aqueous humor of patients with neovascular glaucoma. Ophthalmology 105: 232–237. 9479280 10.1016/s0161-6420(98)92782-8

[5] Sharma PS, Sharma R, Tyagi T (2011) VEGF/VEGFR pathway inhibitors as anti-angiogenic agents: present and future. Curr Cancer Drug Targets 11: 624–653. 21486218 10.2174/156800911795655985

[6] Luttun A, Autiero M, Tjwa M, Carmeliet P (2004) Genetic dissection of tumor angiogenesis: are PlGF and VEGFR-1 novel anti-cancer targets? Biochim Biophys Acta 1654: 79–94. 14984769 10.1016/j.bbcan.2003.09.002

[7] Dewerchin M, Carmeliet P (2012) PlGF: a multitasking cytokine with disease-restricted activity. Cold Spring Harb Perspect Med 2. 10.1101/cshperspect.a011056 PMC3405829 22908198

[8] Carmeliet P, Moons L, Luttun A, Vincenti V, Compernolle V, De Mol M, et al (2001) Synergism between vascular endothelial growth factor and placental growth factor contributes to angiogenesis and plasma extravasation in pathological conditions. Nat Med 7: 575–583. 11329059 10.1038/87904

[9] Kowalczuk L, Touchard E, Omri S, Jonet L, Klein C, Valamanes F, et al (2011) Placental growth factor contributes to micro-vascular abnormalization and blood-retinal barrier breakdown in diabetic retinopathy. PLoS One 6: e17462 10.1371/journal.pone.0017462 21408222 PMC3049767

[10] Rakic JM, Lambert V, Devy L, Luttun A, Carmeliet P, Claes C, et al (2003) Placental growth factor, a member of the VEGF family, contributes to the development of choroidal neovascularization. Invest Ophthalmol Vis Sci 44: 3186–3193. 12824270 10.1167/iovs.02-1092

[11] Mitamura Y, Tashimo A, Nakamura Y, Tagawa H, Ohtsuka K, Mizue Y, et al (2002) Vitreous levels of placenta growth factor and vascular endothelial growth factor in patients with proliferative diabetic retinopathy. Diabetes Care 25: 2352 12453985 10.2337/diacare.25.12.2352

[12] Zaman Y, Rehman AU, Memon AF (2013) Intravitreal Avastin as an adjunct in patients with proliferative diabetic retinopathy undergoing pars plana vitrectomy. Pak J Med Sci 29: 590–592. 24353583 10.12669/pjms.292.3044 PMC3809263

[13] Rinaldi M, Chiosi F, Dell'Omo R, Romano MR, Parmeggiani F, Semeraro F, et al (2013) Intravitreal pegaptanib sodium (Macugen) for treatment of myopic choroidal neovascularization: a morphologic and functional study. Retina 33: 397–402. 10.1097/IAE.0b013e318261a73c 22990315

[14] Krispel C, Rodrigues M, Xin X, Sodhi A (2013) Ranibizumab in diabetic macular edema. World J Diabetes 4: 310–318. 10.4239/wjd.v4.i6.310 24379922 PMC3874491

[15] Ferrara N, Damico L, Shams N, Lowman H, Kim R (2006) Development of ranibizumab, an anti-vascular endothelial growth factor antigen binding fragment, as therapy for neovascular age-related macular degeneration. Retina 26: 859–870. 17031284 10.1097/01.iae.0000242842.14624.e7

[16] Wang X, Sawada T, Kakinoki M, Miyake T, Kawamura H, Saishin Y, et al (2013) Aqueous vascular endothelial growth factor and ranibizumab concentrations after monthly and bimonthly intravitreal injections of ranibizumab for age-related macular degeneration. Graefes Arch Clin Exp Ophthalmol. 10.1007/s00417-013-2505-2 24196779

[17] De Falco S (2012) The discovery of placenta growth factor and its biological activity. Exp Mol Med 44: 1–9. 10.3858/emm.2012.44.1.025 22228176 PMC3277892

[18] Cumurcu T, Bulut Y, Demir HD, Yenisehirli G (2007) Aqueous humor erythropoietin levels in patients with primary open-angle glaucoma. J Glaucoma 16: 645–648. 18091448 10.1097/IJG.0b013e31804a5eb3

[19] Kim YG, Hong S, Lee CS, Kang SY, Seong GJ, Ma KT, et al (2009) Level of vascular endothelial growth factor in aqueous humor and surgical results of ahmed glaucoma valve implantation in patients with neovascular glaucoma. J Glaucoma 18: 443–447. 10.1097/IJG.0b013e3181895e5c 19680051

[20] Semenza GL (1994) Regulation of erythropoietin production. New insights into molecular mechanisms of oxygen homeostasis. Hematol Oncol Clin North Am 8: 863–884. 7852211

[21] Campochiaro PA (2013) Ocular neovascularization. J Mol Med (Berl) 91: 311–321. 23329331 10.1007/s00109-013-0993-5 PMC3584193

[22] Semenza GL (1994) Regulation of erythropoietin production. New insights into molecular mechanisms of oxygen homeostasis. Hematol Oncol Clin North Am 8: 863–884. 7852211

[23] Wang GL, Jiang BH, Rue EA, Semenza GL (1995) Hypoxia-inducible factor 1 is a basic-helix-loop-helix-PAS heterodimer regulated by cellular O2 tension. Proc Natl Acad Sci U S A 92: 5510–5514. 7539918 10.1073/pnas.92.12.5510 PMC41725

[24] Semenza GL (2000) HIF-1: mediator of physiological and pathophysiological responses to hypoxia. J Appl Physiol (1985) 88: 1474–1480. 10749844 10.1152/jappl.2000.88.4.1474

[25] Lowe J, Araujo J, Yang J, Reich M, Oldendorp A, Shiu V, et al (2007) Ranibizumab inhibits multiple forms of biologically active vascular endothelial growth factor in vitro and in vivo. Exp Eye Res 85: 425–430. 17714704 10.1016/j.exer.2007.05.008

[26] (2014) The Neovascular Age-Related Macular Degeneration Database: Multicenter Study of 92 976 Ranibizumab Injections: Report 1: Visual Acuity. Ophthalmology. 10.1016/j.ophtha.2013.11.031 24461586

[27] Wolf S, Balciuniene VJ, Laganovska G, Menchini U, Ohno-Matsui K, Sharma T, et al (2013) RADIANCE: A Randomized Controlled Study of Ranibizumab in Patients with Choroidal Neovascularization Secondary to Pathologic Myopia. Ophthalmology. 10.1016/j.ophtha.2013.10.023 24326106

[28] Papadopoulos N, Martin J, Ruan Q, Rafique A, Rosconi MP, Shi E, et al (2012) Binding and neutralization of vascular endothelial growth factor (VEGF) and related ligands by VEGF Trap, ranibizumab and bevacizumab. Angiogenesis 15: 171–185. 10.1007/s10456-011-9249-6 22302382 PMC3338918

[29] Yang X, Zhang Y, Yang Y, Lim S, Cao Z, Rak J, et al (2013) Vascular endothelial growth factor-dependent spatiotemporal dual roles of placental growth factor in modulation of angiogenesis and tumor growth. Proc Natl Acad Sci U S A 110: 13932–13937. 10.1073/pnas.1309629110 23918367 PMC3752238

